# Influence of intense multidisciplinary follow-up and orlistat on weight reduction in a primary care setting

**DOI:** 10.1186/1471-2296-6-5

**Published:** 2005-01-29

**Authors:** Amiel Feigenbaum, Shmuel Pasternak, Efrat Zusk, Miri Sarid, Shlomo Vinker

**Affiliations:** 1 Department of Family Medicine, Sackler School of Medicine, Tel Aviv University; Tel Aviv, Israel; 2 Primary Care Clinic, Bat Yam, Israel; 3 Department of Physiology and Pharmacology, Sackler School of Medicine, Tel Aviv University, Tel Aviv, Israel; 4 Sarid Institute, Kiriat Haim, Israel

## Abstract

**Background:**

Obesity is the most common health problem in developed countries. Recently, several physicians' organizations have issued recommendations for treating obesity to family physicians, including instructions in nutrition, physical activity and medications. The aim of this study was to examine if effective weight-reducing treatment can be given by a family physician. It compares regular treatment with intensive treatment that include close follow-up and orlistat treatment.

**Methods:**

The study was conducted in three primary care clinics. 225 patients were divided into three groups according to their choice. Group A received a personal diet with fortnightly meetings with the family physician and dietitian and orlistat treatment. Group B received a general diet, monthly meetings with the family physician only and orlistat treatment. Group C received a personal diet, monthly meetings with the dietitian only and no drug treatment. The primary endpoint was reduction of at least 5% of the initial weight during the study period.

**Results:**

A greater percentage of patients in group A achieved their weight reduction goals than in other groups (51%, 13% and 9% in groups A, B and C, respectively, p < 0.001). There was a significant reduction in triglycerides in all groups, a significant reduction of low density lipids (LDL) in groups A and B and no significant difference in high density lipids (HDL) in any group.

**Conclusions:**

Significant weight reduction was obtained in a family physician setting. Further research is needed to evaluate if, by providing the family physician with the proper tools, similar success can be achieved in more clinics.

## Background

Obesity is the most common health problem in developed countries [1]. It is a chronic disease and should be treated as such. Its prevalence is increasing worldwide [2]. In the United States, it is estimated that 64% of the adult population is either overweight or obese with a body mass index (BMI; kg/m^2^) above 25 [3]. The rate of obesity is increasing [4] and has risen by more than 75% in the USA since 1980 [5]. In 2001, the prevalence of obesity (BMI ≥ 30) was 20.9% vs 19.8% in 2000, an increase of 5.6% [6]. In Israel, according to a survey of the Nutrition Department of the Ministry of Health, 55% of adult (ages 25–64) women and 59% of adult men have a BMI above 24.9 [7].

Obesity is associated with increased prevalence of many serious chronic diseases such as diabetes mellitus, hypertension, dyslipidemia, and coronary heart disease [8,9]. It may be responsible for approximately 300,000 deaths in the USA per year [10]. In the Nurses Health Study, the 14-year mortality rate for women with a BMI greater than 32 was more than double that of women with a BMI of less than 19 [11]. Obesity now ranks second only to smoking as a cause of preventable death but, soon, obesity may surpass smoking as the leading cause of preventable death in the USA [12]. In the USA, 19% of deaths from coronary disease and 62% of deaths from diabetes can be attributed to obesity [13]. The risk of death from all causes increases in moderately and severely overweight men and women of all age groups [14].

Diet and exercise have limited effectiveness on long-term maintenance of weight loss [15]. Within five to seven years, 95% of all patients regain the lost weight or more [16]. Pharmacotherapy in combination with a reduced energy diet improves long-term efficacy [17]. Loss of 5–10% of their initial body weight substantially improves the health of obese patients and modifies their cardiovascular risk factors [8,18]. Despite growing information on the pathophysiology of obesity and its high prevalence, obesity and obesity-related diseases are still under-diagnosed and untreated by family physicians [19]. Most family physicians cite lack of time, resources, reimbursement from insurance companies, or knowledge of effective interventions as significant barriers [20].

The intervention of primary physicians during a ten minute physician/patient encounter and telephone consultation with a community dietitian resulted in a significant decrease in the weight of patients [20]. Recently, several physicians' organizations have issued recommendations for treating obesity to family physicians, including instructions in nutrition, physical activity and medications. Such recommendations were based on a number of studies that proved the effectiveness of family physician weight-reduction programs, when based on the readiness of patients to make necessary lifestyle changes and use of appropriate techniques to increase the willingness of the patient to make necessary changes [21-24].

The purpose of this study was to examine if more efficient and effective weight-reducing treatment can be given in the family doctor setting. The study compare a non-pharmacological intervention with drug intervention (orlistat) and compare regular management with more intensive family physician based management..

## Methods

### Study design

The study was conducted in three primary care clinics in an urban area in central Israel. The family physicians who took part in this study participated in 80 hours CME course dealing with obesity treatment in Israel.

The patients were divided into three groups according to their choice. Patients in groups A and B were treated with orlistat at 120 mg TID. Orlistat (Xenical ^®^) is a lipase inhibitor for obesity management that acts by inhibiting the absorption of dietary fats.

The patients in group A received in addition a personal reduced-energy diet and met with a family practitioner and a clinical dietitian once every two weeks. The personal diet was created according to the daily schedule and preferred foods of the individual, emphasizing low-fat foods. The patients received instructions regarding the importance of physical activity and, at each meeting, realistic intermediate goals for achieving two or three small changes in eating habits and physical activity, based on the patient's desires, were determined. The obstacles to change and ways to overcome them were discussed. Support, based on improvements in the patient's health parameters such as an improvement in a blood test, was given.

Some of the patients helped with self-criticism, by keeping food and physical activity diaries and by grading their own goal accomplishments. Patients were taught how to resist temptation and reward themselves for success. We also recruited the support and encouragement of the patient's family. At each meeting, the time of the next meeting was determined and it was emphasized that the most important thing for the patients to do was to attend the meetings, even if their goals were not achieved.

Group B patients were treated with 120 mg orlistat t.i.d., a general formulated reduced-energy diet and follow-up by the family physician once every four weeks for weighing and prescription renewal.

In groups A and B, patients were asked at each meeting if any side effects of orlistat appeared.

Patients in group C were given a personal low-calorie diet, designed according to their preferences, and followed-up by a clinical dietitian once a month.

The prescribed daily caloric intake was equal in the three groups and was 1200 calories per day for women and 1500 calories per day for men.

Before the intervention, every patient received an explanation of the three treatments, the importance of reducing weight, and how excess weight affects their health. All were instructed about the recommended rate of weight loss and a final weight reduction goal of at least 5% of their initial weight within half a year was established. Patients who achieved the 5% reduction goal before the end of the study could choose either to stop the intervention or to complete the study period.

### Patients

Obese (BMI > 30) patients of either sex or patients with a BMI above 27 plus two or more cardiovascular risk factors, aged 20–75 years, were eligible for the study. Patients were excluded if they were pregnant or lactating or if they had any contraindication against using orlistat (chronic malabsorption syndrome, cholestasis, pancreatic disease).

Before the intervention, patients underwent an initial screening that included recording of a complete medical history, measurement of vital signs, body weight and height, and calculation of the BMI. Laboratory analysis included a lipid profile. The readiness of the patient to receive treatment was assessed. At the beginning of the intervention, all participants were in the third stage of readiness ("the preparation stage") according to the Transtheoretical Model of Behavior Change.

An adverse event of any dose of orlistat was considered serious if it resulted in death, was life-threatening, required hospitalization, or resulted in significant disability.

### Efficacy measures

The main measure was weight loss. Each patient was weighed during each meeting. The primary endpoint was reduction of at least 5% of the initial weight during the study period (six months). Achievement of this goal was defined as successful treatment. Another measure was improvement in the lipid profile.

The second lipid profile was done to half of the patients, only those who had dyslipidemia in the first profile had been offered a second profile.

### Statistical analysis

Data on patients' background and weight-reduction results between groups were compared using the Chi square test. Continuous data of the groups, i.e., measurements at the beginning of the study and continuous background data, were compared using one-way tests (ANOVA). When significant statistical differences were found among the three groups, the Tukey Post Hoc test was used to examine the statistical difference between each group of two. ANCOVA was also used and, if there was a difference amongst the groups, the significance (for the measured parameter) was checked using the Bonferonni technique.

## Results

Two hundred and twenty-five patients participated in the study. Their demographic characteristics, history of cardiovascular disease, and initial lipid profile are shown in Table 1. There were no significant differences between groups in average age, initial BMI or gender of the participants. The average length of follow-up and the number of meetings varied among groups. There were no cases of significant side effects that required stopping orlistat treatment of any of the participants.

**Table 1:** Participants' demographic data, initial lipids profile, by treatment group

	Group A	Group B	Group C	P* value
Number of patients	62	112	51	
Age (years +/- SD)	47.3 ± 11	46.8 ± 12	51 ± 9.6	NS
Gender (% female)	71	74	61	NS
Ischemic heart disease (%)	0	4	0	NS
Hypertension (%)	44	51	27	P < 0.05
Diabetes mellitus (%)	9	18	20	NS
Dyslipidemia (%)	16	38	66	P < 0.001
Initial body weight				
Initial body mass index (BMI; kg/m^2 ^+/- SD)	33 ± 3.8	34 ± 4.4	31 ± 3.6	P < 0.01 34 > 31(B > C)
Initial triglycerides (mg/dl, +/- SD)	170 ± 53	184 ± 49	255 ± 205	P < 0.01 170 <255> 183 (A < B > C)
Initial low density lipoproteins (LDL; mg/dl, +/- SD)	150 ± 30	156 ± 36	152 ± 44	NS
Initial high density lipoproteins (HDL; mg/dl, +/- SD)	42 ± 7.0	44 ± 6.7	47 ± 14.9	NS
Average length of treatment (weeks, +/- SD)	13 ± 12.0	9 ± 4.7	23 ± 12	P < 0.001 23 > 13 > 9 (C > A > B)
Number of meetings with physician/dietitian (+/- SD)	4.3 ± 2.0	3.5 ± 1.5	5.2 ± 2.9	P < 0.001 5.2 > 3.5 (C > B)

NS = Not significant.

Group A – Orlistat, a personal reduced-energy diet and a meeting with a family practitioner and a clinical dietitian once every two weeks.

Group B – Orlistat, a general formulated reduced-energy diet and follow-up by the family physician once every four weeks.

Group C – a personal low-calorie diet and follow-up by a clinical dietitian once a month.

* When significant statistical differences were found among the three groups, we examined the statistical difference between each group of two.

Patient-reported adverse events in the orlistat-treated groups were all related to the gastrointestinal tract. The most commonly reported events were flatulence with discharge (9.2%), fatty or oily stool with increased defecation (9.2%), feeling of fullness in the stomach (4.6%), and constipation (1.7%). There were no statistically significant differences in side effects between groups A and B. In group A, 11 patients (17.7%) stopped the treatment for the following reasons: cost of the medication (47%), lack of time (33%), or dissatisfaction (20%). In comparison, ten patients (8.9%) in group B stopped the treatment, mainly because of the cost of the treatment (65%) or low motivation (23.5%). The reasons for stopping treatment significantly differed between groups A and B (*p *= 0.03).

**Figure 1:**
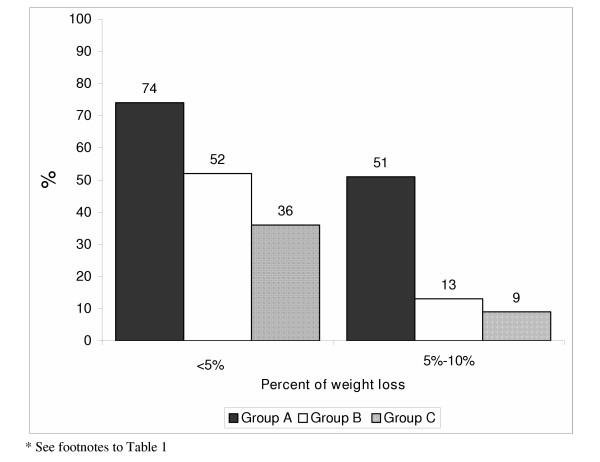
Weight loss by treatment group*

The percentage of patients who attained their weight reduction goals was largest in group A where patients received orlistat and intense follow-up, in addition to a personally-designed diet (Fig. 1). Changes in lipid profiles are shown in Table 2. The treatment resulted in a significant reduction in triglyceride levels in all groups, a significant reduction of low density lipoproteins (LDL) in groups A and B and no significant difference between initial and final high density lipoproteins (HDL) in any group.

**Table 2:** Changes in lipid profile during treatment according to treatment group^1^(paired sample *t *test)

	Group A	Group B	Group C
Number of patients^2^	32	55	28
Initial triglycerides (mg/dl, +/- SD)	170 ± 53	184 ± 49	255 ± 205
Final triglycerides (mg/dl, +/- SD)	139 ± 43	153 ± 35	165 ± 60
Delta (+/- SD)	-31 ± 21	-31 ± 25	-90 ± 187
P value (pre-post)	<0.001	<0.001	0.01
Initial low density lipids (LDL; mg/dl, +/- SD)	150 ± 30	156 ± 36	152 ± 44
Final LDL (mg/dl, +/- SD)	129 ± 28	143 ± 32	147 ± 34
Delta	-21 ± 26	-12 ± 16	-5 ± 34
P value (pre-post)	<0.001	<0.001	NS
Initial high density lipids (HDL; mg/dl, +/- SD)	42 ± 7.0	44 ± 6.7	47 ± 14.9
Final HDL (mg/dl, +/- SD)	43 ± 6.6	45 ± 6.7	48 ± 15.3
Delta (+/- SD)	0.9 ± 2.9	0.8 ± 3.3	1.4 ± 8.8
P value (pre-post)	NS	NS	NS

NS = Not significant.

^1 ^See footnotes to Table 1

^2 ^Patients with pre and post lipid profile analyses

Patients in Group A reduced 5.12 kg (range of 5–8 Kg.) of their initial body weight, patients in Group B reduced 7.8 kg (range 10–12 Kg.) from their initial body weight and patients in Group C reduced 3.12 kg (range 5–6 Kg.) of their initial body weight.

## Discussion

Obesity is a worldwide problem. Treatment of a patient for obesity involves two processes: evaluation of the severity of the obesity and general health condition of the patient, and management which includes guidance in how to gradually reduce weight and maintain the new weight together with imparting healthy lifestyle habits and keeping track of improvement.

In this study, we examined weight-reduction techniques that can be carried out in the setting of a community family practice. Intense treatment, combining frequent counseling by the family physician and a dietitian with medications (group A), resulted in the best weight reduction and lipid profile improvement in the short period of this study, as was reported earlier in special weight reduction clinics [25-27]

The effectiveness of interventions in primary care setting are controversial [28-30]. Beermann et al [28] in a community survey of 792 patients found that Orlistat was not prescribed according to the approved indication in the majority of cases. The dropout rate was high and most patients had minor gain from the treatment. Linne et al [29] noted that success rate of Orlistat in primary-care practice is limited by failure to follow prescribing recommendations. A simple questionnaire to 70 patients revealed that in many cases the referral physician had not observed basic rules and regulations, nor given appropriate information on Orlistat use.

Hauptman et al [30] in a study conducted in seventeen primary care centers in the United States. The study indicates that orlistat is an effective adjunct to dietary intervention in the treatment of obesity in primary care settings.

There are many advantages to a program involving cooperation between the family physician and the dietitian. The family physician is acquainted with the patient for a longer time than a dietitian. The physician is familiar with the patient's health condition, medications taken by the patient, the patient's environment and lifestyle and can recommend a treatment suitable to the patient's personality and lifestyle. The family physician is knowledgeable about weight-reducing drugs and possible side effects. The patient trusts and has confidence in the family doctor. Together with the dietitian, a personal diet appropriate to the drug treatment can be created.

Obesity is a chronic disease. The physician and the patient must recognize that obesity treatment is a prolonged process that extends a lifetime. Since family physicians are usually familiar with their patients as well as with the patient's family and environment for many years, family physicians know what changes the patient can achieve. They can recruit family members to support the necessary lifestyle changes. Also, because of their training, family physicians are the most suitable professional to holistically treat obesity and its complications.

There were several limitations in our study. One limitation was the non-random division of patients into groups A, B and C. It is possible that the patients who chose drug treatment differed from those who chose a diet only. Perhaps they were more ready for the weight-reduction process and to expend money for the drug (in Israel, there is a patient co-payment for medications). The rate of women amongst the patients seeking treatment was higher than the rate of men, as in other reports [26,27]. Hence, we assume that our study represented the segment of the population more prone to try dieting and weight-reduction programs. Ways to increase the number of men participating in weight-reduction programs must be found.

The study periods for groups A and B (which received orlistat) were shorter than for group C, partly because there is significant co-payment for orlistat and partly because the targeted weight had been achieved earlier. The drug was well tolerated with minimal gastrointestinal side effects.

The patients were treated by family physicians that were orientated toward and trained in weight reduction. Results might have been less successful with other physicians. Hence, family physicians should be trained in the subject by increasing both their knowledge and their treatment skills. Healthcare funds must recognize the importance of weight reduction so that they will allocate the necessary additional time and resources of their physicians, clinics and multi-field staff.

This study evaluated only the weight reduction period. Long-term results and whether or not the patient maintained the lifestyle change for a long period were not examined. Further studies should examine the best program for maintaining the new weight.

In conclusion, this study showed that within the setting of the family practice it is possible to carry out an effective program of weight reduction and achieve significant weight loss. The addition of orlistat can further improve results. We believe that by providing family physicians with the proper tools, similar success can be achieved in many more clinics.

## Competing interests

The author(s) declare that they have no competing interests.

## Authors' contributions

AF and SP conceived and designed the study, participated in the collection, analysis and interpretation of data and drafted the manuscript. SV Participated in the statistical analysis, interpretation of data and draft of the manuscript. MS and EF participated in the design of the study, data collection and interpretetion. All authors read and approved the final manuscript.

## Pre-publication history

The pre-publication history for this paper can be accessed here:


